# First-in-Human Dose Selection, Pharmacokinetics Prediction, and Clinical Validation of SYHA1805, a Novel FXR Agonist, Using Allometric Scaling and PBPK Modeling

**DOI:** 10.3390/pharmaceutics18070862

**Published:** 2026-07-15

**Authors:** Lei Zhang, Miao Zhang, Enda Zhou, Xueyuan Zhang, Huanhuan Qi, Xueting Yao, Dongyang Liu

**Affiliations:** 1 Drug Clinical Trial Center, Department of Pharmacy, Peking University Third Hospital, Beijing 100191, China; zhanglei_bjmu@163.com (L.Z.); zz_mm_zz@163.com (M.Z.); zhouenda_1133@163.com (E.Z.); 2 Center of Clinical Medical Research, Institute of Medical Innovation and Research, Peking University Third Hospital, Beijing 100191, China; 3 Department of Pharmaceutical Sciences, School of Pharmacy, Bouvé College of Health Sciences, Northeastern University, Boston, MA 02115, USA; 4 CSPC Zhongqi Pharmaceutical Technology (SJZ) Co., Ltd., Shijiazhuang 050035, China; zhxueyuan@cspc.cn (X.Z.); qhuanhuan@cspc.cn (H.Q.)

**Keywords:** SYHA1805, metabolic dysfunction-associated steatohepatitis, FXR agonist, PBPK modeling, first-in-human, allometric scaling

## Abstract

**Background:** SYHA1805 is a potent farnesoid X receptor (FXR) agonist currently in development for Metabolic Dysfunction-Associated Steatohepatitis (MASH). **Methods:** To determine the first-in-human (FIH) dose and guide its clinical development, an integrated approach combining in vitro and in vivo ADME and toxicological characterizations, cross-species allometric scaling (AS), and physiologically based pharmacokinetic (PBPK) modeling was employed. **Results:** Using monkeys and rats as extrapolation species, AS predicted a human intravenous clearance of 20.7 L/h and a steady-state volume of distribution of 15.1 L. Based on body surface area and exposure-based modeling, an FIH dosing regimen for single-dose administration was proposed, ranging from a 30 mg starting dose to a 3000 mg maximum, with an effective dose of 1150 mg. These dosing strategies were further supported by PBPK models, which accurately estimated human systemic exposure. The model simulations were subsequently validated by clinical trial data from a single ascending dose (SAD) study (CTR20202354). **Conclusions:** These findings establish a robust pharmacokinetic foundation for the continued clinical advancement of SYHA1805.

## 1. Introduction

Metabolic dysfunction–associated fatty liver disease (MAFLD) affects more than 25% of the global population and represents a growing public health burden. Its disease spectrum spans from metabolic dysfunction-associated fatty liver (MAFL) to metabolic dysfunction-associated steatohepatitis (MASH), cirrhosis, and hepatocellular carcinoma (HCC) [1]. Approximately 10–30% of individuals with MAFLD progress to MASH, a transition associated with a more than 11-fold increased risk of HCC compared with simple steatosis [2]. In recent years, MASH has become one of the leading indications for liver transplantation in the United States and Europe [3], and affected individuals experience substantial post-transplant morbidity and early mortality, driven in part by cardiovascular complications [4]. The disease is tightly linked to systemic metabolic dysfunction, affecting 40–70% of individuals with type 2 diabetes and 60–90% of those with obesity [5,6]. Collectively, its high prevalence, progressive nature, and multisystem comorbidities establish MASH as a critical unmet medical challenge.

Despite increasing therapeutic interest, treatment options for MASH remain limited. The recent approval of resmetirom [7] and semaglutide [8] marks important progress; however, their efficacy, safety, and accessibility leave significant unmet needs [9]. The farnesoid X receptor (FXR), a bile acid-activated nuclear receptor, has emerged as a compelling therapeutic target due to its central role in regulating bile acid, lipid, and glucose homeostasis [10]. FXR activation suppresses hepatic gluconeogenesis and lipogenesis, improves insulin sensitivity, and modulates inflammatory and fibrotic pathways, supporting its potential in MASH treatment [11]. Obeticholic acid, the most clinically advanced FXR agonist, has not been approved for MASH by the U.S. Food and Drug Administration (FDA) following phase 3 evaluation, largely due to safety concerns, including severe pruritus, dyslipidaemia, and gallstone formation [12,13]. These adverse effects are thought to reflect limitations in receptor selectivity and pharmacokinetic properties [14], including hepatic accumulation associated with enterohepatic circulation. Such challenges have driven the development of non-steroidal FXR agonists, which may offer improved selectivity and more favorable safety profiles [15,16,17]. Currently, several such compounds, including Tropifexor and Cilofexor, are in various stages of clinical development [16,18].

SYHA1805 (chemical structure shown in Supplementary Figure S1) is a novel non-steroidal FXR agonist with high potency and marked selectivity over Takeda G protein-coupled receptor 5 (TGR5) (>7720-fold, based on in-house EC_50_ determinations for FXR and TGR5 activation using in vitro and cell-based assays). TGR5 is a G protein-coupled receptor (GPCR) that is naturally activated by bile acids. Although literature has reported its potential therapeutic benefits in fatty liver disease and diabetes, systemic activation of TGR5 may lead to adverse effects, including gallbladder toxicity, pruritus, and potential cardiovascular side effects [19]. Therefore, avoiding off-target activation of TGR5 is of great significance for reducing the risk of potential adverse effects.

For such first-in-class candidates, a comprehensive understanding of absorption, distribution, metabolism, and excretion (ADME) properties is essential to inform clinical translation. First-in-human (FIH) dose selection remains a critical step in drug development, requiring a careful balance between patient safety and development efficiency. Regulatory agencies, including the National Medical Products Administration (NMPA), the FDA, and the European Medicines Agency (EMA), emphasize data-driven strategies informed by preclinical pharmacology and toxicology [20,21,22]. Established approaches such as no-observed-adverse-effect level (NOAEL), minimal anticipated biological effect level (MABEL), pharmacokinetic-based scaling, and pharmacokinetic/pharmacodynamic modelling each provide complementary insights but also have inherent limitations, necessitating integrated evaluation frameworks [23,24,25,26,27].

Here, we present a comprehensive translational assessment of SYHA1805 to support its clinical development. By integrating in vitro ADME characterization, cross-species pharmacokinetic analysis, toxicological thresholds, and physiologically based pharmacokinetic (PBPK) modelling, we established a framework for predicting human exposure and informing FIH dose selection. The agreement between model-based predictions and subsequent clinical observations supports the robustness of this approach. These findings not only advance the clinical evaluation of SYHA1805 but also provide a generalizable strategy for the development of non-steroidal FXR agonists in MASH.

## 2. Materials and Methods

### 2.1. Materials

Acetonitrile (Fisher Scientific, Waltham, MA, USA). Formic acid (FA) and ammonium acetate (NH_4_OAc) (Merck, Darmstadt, Germany). Caco-2 cells (American Type Culture Collection, Manassas, VA, USA). Liver microsomes from CD-1 mice, Sprague–Dawley (SD) rats, and beagle dogs (Xenotech, Kansas City, MO, USA). Liver microsomes from cynomolgus monkeys and humans (Corning, Woburn, MA, USA). Hepatocytes from humans, beagle dogs, and SD rats (BioIVT, Baltimore, MD, USA). Hepatocytes from cynomolgus monkeys and CD-1 mice (Xenotech, Kansas City, MO, USA). Plasma from CD-1 mice, SD rats, beagle dogs, and humans (mixed sex) (BioIVT, Baltimore, MD, USA). Plasma from cynomolgus monkeys (Suzhou Xishan Zhongke Experimental Animal Co., Ltd., Suzhou, China). SD rats (Beijing Vital River Laboratory Animal Technology Co., Ltd., Beijing, China, certificate no. 11400700385648). Cynomolgus monkeys (Hainan Primate Research Center, Haikou, China, license no. SCXK (Qiong) 2016-0004). Male C57BL/6J mice (Nanjing Biomedical Research Institute of Nanjing University, Nanjing, China, certificate no. 201802553). All animal studies and clinical procedures were conducted in accordance with approved ethical guidelines and relevant regulations.

### 2.2. Species Selection for Extrapolation

To facilitate species extrapolation, the metabolic stability and metabolite profile of SYHA1805 were characterized across relevant species. Metabolic stability was assessed in liver microsomes from CD-1 mice, SD rats, beagle dogs, cynomolgus monkeys, and humans (n = 3 per species). In parallel, metabolite identification was conducted using both liver microsomes and cryopreserved hepatocytes (n = 1 per species). Microsomal incubations were initiated with NADPH and terminated at defined intervals, whereas hepatocyte incubations were performed at 37 °C under 5% CO_2_ for 24 h. All reactions were quenched with acetonitrile. The percentage remaining, half-life (T_1/2_), intrinsic clearance (CL_int_), and hepatic clearance (CL_hep_) were calculated using Equations (S1)–(S4) in Supplementary Materials, with the required parameters provided in Supplementary Table S1.

### 2.3. Determination of Parameters for Extrapolation and PBPK Model

#### 2.3.1. Physicochemical Parameters, Permeability, and Plasma Protein Binding

The pKa of SYHA1805 was determined using potentiometric titration/UV-visible spectroscopy, and the oil/water partition coefficient (logP) of the compound was measured using the shake flask method with n-octanol/buffer solution.

The bidirectional permeability and efflux ratio of SYHA1805 were determined using the Caco-2 cell monolayer. Caco-2 cells were seeded onto a 96-well plate and cultured for twenty-six days. The Monolayer integrity was confirmed by measuring transepithelial electrical resistance (TEER) values (>400 Ω·cm^2^). SYHA1805 at concentrations of 2, 10, and 100 µM was then added to the apical or basolateral compartments of the culture chamber with triplicates prepared for each concentration. After incubating at 37 °C for 120 min, the incubation media from the apical and basolateral sides were collected. The effect of P-glycoprotein (P-gp) on the absorption of SYHA1805 was examined in the presence or absence of GF120918, a classic P-gp inhibitor with an IC_50_ of 2 μM in Caco-2 cells [28]. During the incubation, the Lucifer yellow rejection assay was also performed to further confirm the integrity of the monolayer. P_app_ and the efflux ratio were calculated using Equations (S5) and (S6).

Plasma protein binding was determined by equilibrium dialysis in five species at concentrations of 0.2, 2, and 10 μM (triplicate per concentration). The unbound fraction was calculated from buffer and plasma concentrations after 4 h of dialysis.

#### 2.3.2. Pharmacokinetic Parameters in SD Rats and Cynomolgus Monkeys

All animal experimental protocols were approved by the Institutional Animal Care and Use Committee (IACUC) of WuXi AppTec (Shanghai, China). All animal husbandry, management, euthanasia, and experimental procedures were conducted in accordance with the standards and principles of the Association for Assessment and Accreditation of Laboratory Animal Care (AAALAC). The sample size was minimized while ensuring sufficient statistical power for all animal experiments. Animals were randomly assigned to the control and treatment groups by stratified randomization according to body weight. Pharmacokinetic studies were conducted in SD rats and cynomolgus monkeys (n = 24 per species; 4 groups, 3/sex/group). Animals received a single intravenous dose (2 mg/kg for rats; 1 mg/kg for monkeys) or oral doses of 10, 30, or 100 mg/kg. At each time point, blood samples of 0.1 mL (rats) and 1.0 mL (monkeys) were collected according to the following schedule: for rats, pre-dose and 0.083 (intravenous only), 0.25, 0.5, 1, 2, 4, 6, 8, 24, 32, and 48 h post-dose; for monkeys, pre-dose and 0.083 (intravenous only), 0.25, 0.5, 1, 2, 4, 6, 8, 10, and 24 h post-dose. Plasma was separated from whole blood by centrifugation at 3000× *g* at 4 °C for 15 min, immediately snap-frozen on dry ice, and stored at −80 °C until analysis.

Concurrently, tissue distribution studies were performed in SD rats (n = 24; 4 groups, 3/sex/group). Animals received a single oral dose of 30 mg/kg SYHA1805 and were euthanized at 0.5, 2, 24, and 72 h post-dose. Whole blood was collected, followed by whole-body perfusion with saline via the heart. Thirteen tissues were harvested (heart, liver, spleen, lung, kidney, stomach, small and large intestine, muscle, fat, brain, and sex-specific organs: uterus and ovary for females; prostate and testis for males).

For sample preparation, plasma samples (30 µL) were mixed with 270 µL of internal standard (IS) working solution (10.0 ng/mL tolbutamide in acetonitrile) for protein precipitation. The mixture was vortexed for 15 min and then centrifuged at 3220× *g* at 4 °C for 15 min. An aliquot of 100 µL supernatant was diluted with an equal volume of water, vortexed for 5 min, and centrifuged again at 3220× *g* at 4 °C for 5 min. The resulting supernatant was subjected to LC-MS analysis. Tissue samples were homogenized in 9 volumes of ice-cold homogenization buffer (methanol/phosphate-buffered saline, pH 7.4, 1:2, *v/v*). Then, 50 µL of tissue homogenate was mixed with 200 µL of IS working solution for protein precipitation. The subsequent procedure was the same as that described for plasma samples.

Drug concentrations in biological samples were analyzed by LC-MS to determine pharmacokinetic parameters. The LC system consisted of a Waters ACQUITY UPLC system. Mobile phase A was a solution containing 0.1% FA and 2 mmol/L NH_4_OAc in water and acetonitrile (*v/v*, 95:5), and mobile phase B was a solution containing 0.1% FA and 2 mmol/L NH_4_OAc in water and acetonitrile (*v/v*, 5:95). Gradient elution was performed with the following program: 0–0.1 min, 30% B; 0.1–1.2 min, 30–100% B; 1.2–1.5 min, 100% B. The flow rate was 0.6 mL/min. Chromatographic separation was achieved on an ACQUITY UPLC BEH C18 column (2.1 × 50 mm, 1.7 µm; Waters, Milford, MA, USA) maintained at 60 °C. The autosampler temperature was set at 10 °C, and the injection volume was 10 µL.

Mass spectrometric detection was performed on an AB SCIEX Triple Quad 6500+ mass spectrometer operating in positive electrospray ionization (ESI) mode with multiple reaction monitoring (MRM). The ion source parameters were as follows: ion spray voltage, 5000 V; source temperature, 500 °C; nebulizer gas (GS1), 50 psi; heater gas (GS2), 50 psi; curtain gas (CUR), 35 psi; and collision gas (CAD), 10 psi. The MRM transition for SYHA1805 was m/z 584.0 → 266.2, with a dwell time of 70 ms, declustering potential (DP) of 92 eV, and collision energy (CE) of 32 eV.

In accordance with the Technical Guidelines for Non-clinical Pharmacokinetic Studies issued by the National Medical Products Administration (NMPA) and the International Council for Harmonisation of Technical Requirements for Pharmaceuticals for Human Use (ICH) M10 guideline for Bioanalytical Method Validation and Study Sample Analysis, the analytical method was fully validated with respect to linearity, intra- and inter-batch precision and accuracy, sensitivity, specificity, matrix effect, dilution integrity, extraction recovery, carryover, system suitability, reinjection reproducibility of processed samples, and stability in biological samples.

#### 2.3.3. Toxicity Studies

Acute and repeated-dose toxicity studies were conducted in SD rats and cynomolgus monkeys with appropriate vehicle controls. In rats, acute toxicity was assessed at 200–2000 mg/kg (n = 8 per group, equal sex distribution), and repeated dosing (100–1000 mg/kg, n = 30 per group, equal sex distribution) was conducted for 28 days followed by a 4-week recovery period. In monkeys, acute toxicity was evaluated at 100–1000 mg/kg (n = 2 per group, equal sex distribution) and repeated dosing at 50–500 mg/kg (n = 10 per group, equal sex distribution) for 28 days with recovery. Endpoints included clinical observations, body weight, food intake, ophthalmology, clinical pathology, organ weights, and histopathology. Additional endpoints in monkeys included electrocardiography and body temperature. NOAEL and MTD values were subsequently determined.

### 2.4. The Pharmacodynamics of SYHA1805 in Mice MASH Model

A mouse MASH model was prepared by high-fat diet combined with CCl_4_ injection. Different doses of SYHA1805 (7.5, 15, and 45 mg/kg, n = 8 per group, male) were administered by gavage. In addition, solvent control in the model group and healthy group were prepared in parallel. The animals were euthanized after 28 days of continuous administration with SYHA1805. Liver pathological examinations were performed with Hematoxylin and Eosin staining and Sirius Red staining, and the severity of MASH was quantified using the NAFLD Activity Score (NAS). The NAS is the gold-standard histological scoring system that integrates the evaluation of steatosis, lobular inflammation, and hepatocellular ballooning [29].

### 2.5. Prediction of First-in-Human Dose

Human PK parameters and the FIH starting dose of SYHA1805 were estimated using allometric scaling approaches in accordance with regulatory guidance from the FDA and the NMPA. Human intravenous clearance (CL_i.v,h_) was predicted using multiple complementary methods, including single-species scaling, body weight-based allometric scaling with plasma protein binding correction, the *f*u-corrected intercept method, and the hepatic blood flow method (Equations (S7)–(S10)) [30,31]. The steady-state volume of distribution in humans (V_ss,h_) was predicted using allometric scaling (Equation (S11)) from animal values (V_ss,a_) with scaling coefficients of 1 for rats and monkeys [31,32].

The maximum recommended starting dose (MRSD) and human equivalent dose (HED) were subsequently estimated based on NOAELs obtained from repeat-dose toxicity studies in SD rats and cynomolgus monkeys. Two complementary approaches were applied. In the exposure-based method, HED was calculated using animal AUC_0–24h_, adjusted for interspecies differences in plasma protein binding and scaled by predicted human clearance and animal bioavailability (Equation (S12)), and MRSD was obtained by applying a safety factor (SF) of 100 (Equation (S13)). In parallel, the body surface area-based method was used, in which HED was derived from NOAEL values using species-specific conversion coefficients (K = 0.16 for rats and 0.32 for monkeys) and human body weight (Equation (S14)). Together, these approaches provided a conservative and mechanistically informed estimation of the initial clinical dose and safety margins for SYHA1805.

### 2.6. PBPK Modeling

#### 2.6.1. Model Development in Rat, Monkey, and Human

PBPK models of SYHA1805 were sequentially developed for rats, cynomolgus monkeys, and humans. Model development followed a stepwise, bottom-up approach integrating in vitro data, preclinical pharmacokinetics, and interspecies scaling. An intravenous rat PBPK model was first established using a full-PBPK structure. Tissue distribution was described using predicted V_ss_ and the tissue-to-plasma partition coefficient (K_p_), with a K_p_ scalar optimized to align simulated V_ss_ with observed values. Experimentally determined CL_i.v,rat_ was directly incorporated. In the absence of measured blood-to-plasma (B/P) ratios across species, the rat B/P ratio was estimated by fitting the intravenous plasma concentration–time profile and subsequently applied across species. An oral rat PBPK model was then developed using the same distribution parameters. Absorption was described using a first-order model informed by permeability data from the Caco-2 system, with apparent permeability (P_app_) calibrated against the reference compound metoprolol [33]. The absorption rate constant (k_a_) was estimated by fitting pharmacokinetic data at 10 mg/kg and subsequently verified using data at 30 and 100 mg/kg.

Monkey PBPK models for both intravenous and oral administration were developed by adapting the rat model structure, with species-specific K_p_ and CL_i.v,monkey_ values derived from experimental data. B/P ratios were assumed to be conserved across species. Model performance was evaluated by comparing simulated and observed pharmacokinetic profiles following intravenous and oral dosing in monkeys. The human PBPK model was subsequently established using extrapolated parameters, including CL_i.v,h_, K_p_ (derived from predicted V_ss_ via allometric scaling), k_a_, and the cross-species B/P ratio. Physicochemical and biochemical parameters, including molecular weight, plasma protein binding, logP, and pk_a_, were obtained from experimental measurements and incorporated into the model.

#### 2.6.2. Model Simulation and Validation

The human PBPK model was used to predict systemic exposure of SYHA1805 in healthy adult volunteers (20–50 years) based on the proposed clinical dosing regimen. Simulations were conducted using a virtual population (10 trials of 10 subjects each; n = 100) with equal representation of males and females.

Model validation was performed using data from a single ascending dose (SAD) clinical study (CTR20202354). The simulated trial design matched the clinical protocol, including demographic characteristics, dosing conditions, and sampling schedules. Predictive performance was evaluated using two predefined criteria: (i) observed concentration–time profiles falling within the 90% confidence interval of model predictions; and (ii) ratios of key pharmacokinetic parameters (AUC, C_max_, and CL/F) within 0.5–2.0-fold of observed values.

### 2.7. Statistical Analysis

No animals or data points were excluded from the analysis. Continuous data are presented as mean ± standard deviation (SD). Pharmacokinetic parameters of SD rats, cynomolgus monkeys, and humans were calculated using non-compartmental analysis (NCA) with WinNonlin software (version 8.2). In the mouse MASH model, liver histopathological scoring was performed in a blinded manner, and group comparisons were analyzed by one-way analysis of variance (ANOVA) with appropriate post hoc tests. Statistical significance was set at *p* < 0.05. PBPK model development and simulations were performed using the Simcyp Simulator (version 20; Certara).

## 3. Results

**Figure 1:**
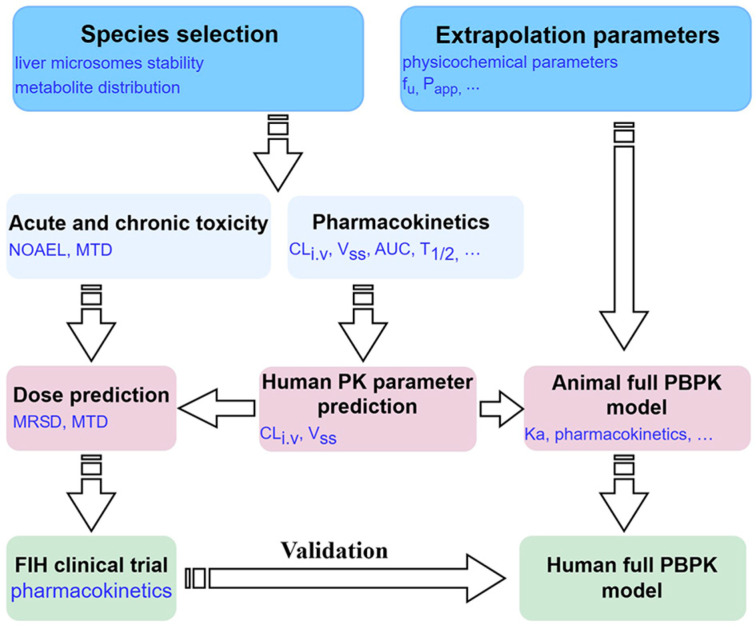
Overall strategy for the prediction of the first-in-human dose and pharmacokinetics of SYHA1805.

To clearly illustrate the overall strategy of this study, we constructed a workflow diagram as shown in Figure 1. The process began with systematic preclinical studies to identify extrapolation species and generate key parameters. These data were then integrated to perform interspecies extrapolation of pharmacokinetics and to establish and optimize animal and human PBPK models. The human model was subsequently validated using FIH clinical trial data. Ultimately, a validated human PBPK model was established to support clinical dose selection and development decisions.

### 3.1. In Vitro Metabolism, Physicochemical Properties, and Absorption Characteristics

The metabolic stability, metabolite profile, physicochemical properties, absorption, and plasma protein binding of SYHA1805 were firstly evaluated across species. In liver microsomes, SYHA1805 exhibited time-dependent metabolic depletion over 60 min, with rapid clearance observed in rodents (near-complete metabolism within 20 min), moderate metabolism in cynomolgus monkeys, and comparatively slower and similar metabolic profiles in beagle dogs and humans (Figure S2 and Table S2), indicating marked interspecies differences in intrinsic and hepatic clearance. Metabolite profiling in liver microsomes and hepatocytes identified 12 metabolites, with extensive biotransformation in rodents and a metabolic pattern in cynomolgus monkeys most closely resembling that in humans (Tables S3 and S4), supporting the selection of SD rats and cynomolgus monkeys for subsequent translational studies. Physicochemical characterization showed that SYHA1805 has a pk_a_ of 2.95 and a logP of 1.63, consistent with moderate lipophilicity. In Caco-2 assays, SYHA1805 demonstrated low permeability and a high efflux ratio (>2), which was markedly reduced in the presence of the P-gp inhibitor GF120918 (Table 1), indicating that active efflux transport contributes to its limited intestinal absorption. In addition, SYHA1805 exhibited consistently high plasma protein binding (>98%) across all tested species without concentration dependence (Table S5), suggesting a low unbound fraction in systemic circulation.

### 3.2. Pharmacokinetics and Toxicity Evaluation in Rats and Monkeys

The analytical method for SYHA1805 was systematically optimized and validated. The plasma validation results demonstrated good linearity over the concentration range of 1.00–1000 nM (R^2^ ≥ 0.9926). The intra- and inter-batch precision and accuracy for four quality control (QC) samples at different concentrations (n = 6) met the acceptance criteria, with absolute deviations from nominal values ≤ 7.3% and CV ≤ 11.4%. The extraction recovery at all concentrations was ≥82.0%, with a CV≤ 5.3%. No endogenous interference was observed in blank matrices from six different sources, and no significant matrix effect was detected. For high-concentration samples, the deviation from nominal values was ≤8.0% under 10-fold dilution. Carryover was below 20.0% of the peak area of the lower limit of quantification (LLOQ) sample. Moreover, the absolute deviations from nominal values for reinjection reproducibility, stability at room temperature for 24 h, stability at −80 °C for 15 days, and freeze–thaw stability (5 cycles) in plasma were within 8.6%. All these results met the requirements of relevant guidelines, indicating that the method is suitable for the bioanalysis of SYHA1805. Additionally, as with the plasma assay, the analytical method for tissue samples was also fully validated in compliance with regulatory guidance requirements, thereby ensuring the reliability and acceptability of the generated tissue distribution data.

Plasma concentrations of SYHA1805 were subsequently analyzed, with the resulting concentration–time curves presented in Figure S3. Pharmacokinetic parameters in SD rats and cynomolgus monkeys were derived by NCA. Following intravenous administration, SYHA1805 exhibited rapid clearance and moderate distribution in SD rats (2 mg/kg; CL_i.v,rat_ 80.9 ± 20.4 mL/min/kg; V_ss,rat_ 1.78 ± 0.49 L/kg; T_1/2,rat_ 1.11 ± 0.20 h), whereas cynomolgus monkeys (1 mg/kg) showed lower clearance and smaller distribution volume (CL_i.v,monkey_ 8.35 ± 2.20 mL/min/kg; V_ss,monkey_ 0.216 ± 0.109 L/kg; T_1/2,monkey_ 3.30 ± 1.60 h), resulting in higher systemic exposure (Table 2). After oral administration (10–100 mg/kg), SYHA1805 was rapidly absorbed in rats, with dose-proportional increases in C_max_ and exposure. Gender-related differences were observed, with higher exposure in females (Table S6). In contrast, cynomolgus monkeys exhibited delayed absorption (prolonged T_max_) and very low oral bioavailability (~1%), despite a dose–exposure relationship. No significant gender differences were observed in monkeys.

Tissue concentrations in SD rats were also measured at designated time points, with distribution profiles presented in Figure S4. Peak exposure across all organs occurred at 0.5–2 h. Sex-based analysis revealed that male rats had higher exposure in the stomach, whereas females showed greater exposure in the large intestine, small intestine, liver, fat, and muscle. In both sexes, the gastrointestinal tract and liver were the primary exposure organs, with the liver being the major enrichment site following absorption.

No mortality was observed during the toxicity study. Gastrointestinal reactions and alterations in biochemical parameters were noted in the medium- and high-dose groups in both rats and monkeys. Notably, only alkaline phosphatase (ALP) showed an approximately 2-fold increase in the high-dose groups, whereas no significant changes were observed in alanine aminotransferase (ALT), aspartate aminotransferase (AST), or other parameters. Moreover, no obvious histopathological changes were detected in the liver, kidney, or other organs. Most of these parameters returned to normal levels by the end of the recovery period. Based on toxicity profiles, the NOAEL was determined to be 1000 mg/kg in SD rats, and no MTD was reached. In cynomolgus monkeys, the NOAEL and MTD were determined to be 150 mg/kg and 500 mg/kg, respectively. As most adverse findings were reversible, these NOAELs provided a sufficient safety margin to support subsequent clinical studies.

### 3.3. Pharmacodynamics Results of SYHA1805 in High-Fat Diet and CCl_4_-Induced Mouse Model

**Figure 2:**
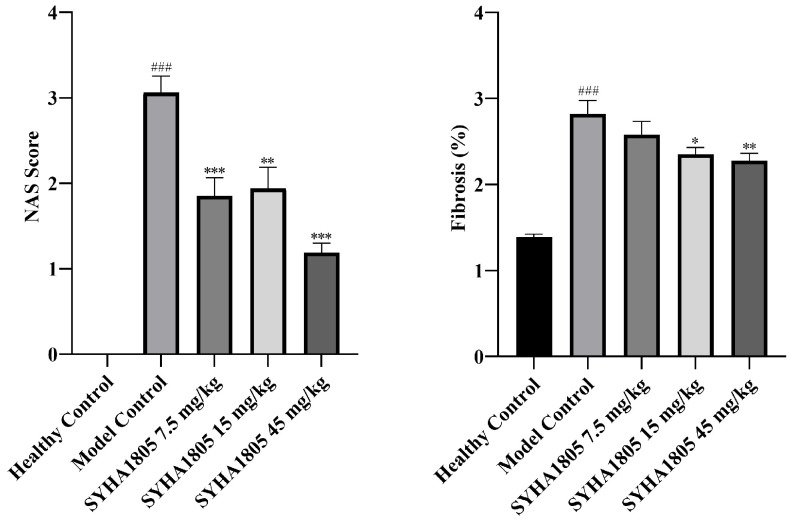
The NAS and fibrosis scores of MASH model mice after treatment with different doses of SYHA1805 (n = 8 per group). ### *p* < 0.001 compared with the healthy control, * *p* < 0.05, ** *p* < 0.01, *** *p* < 0.001 compared with the model control.

The staining results were analyzed, and the NAS and fibrosis scores are shown in Figure 2. From the results, different doses of SYHA1805 could significantly reduce the NAS score and improve the fibrosis, although no significant difference was observed with low dose in fibrosis improvement. According to the FDA guidelines for MASH therapeutic drug development, a dose of 7.5 mg/kg was selected as the minimum effective dose in mice. It is worth mentioning that at this dose, both liver total cholesterol (TC) and triglyceride (TG) showed significant improvement.

### 3.4. Predicted Human Pharmacokinetic Parameters and First-in-Human Dose

Human pharmacokinetic parameters were predicted using allometric scaling based on rat and monkey data. Predicted CL_i.v,h_ differed between species (Table S7), with estimates of 52.5 L/h (rat-based) and 20.7 L/h (monkey-based), reflecting interspecies variability. Given the closer metabolic similarity between cynomolgus monkeys and humans, monkey-derived parameters were prioritized. The predicted V_ss,h_ (70 kg) was 15.1 L (range 15.1–125 L). Based on toxicity data and conservative safety considerations, the HED derived from monkey studies was 3360 mg. A starting dose of 30 mg was selected for FIH evaluation, with a projected MTD of approximately 3000 mg (Table S8). Meanwhile, based on the MABEL dose determined in the pharmacodynamic experiments in mice, the predicted effective dose was 1150 mg, which falls into the dose range set for the current FIH trial.

### 3.5. PBPK Model Simulation and Clinical Validation

**Figure 3:**
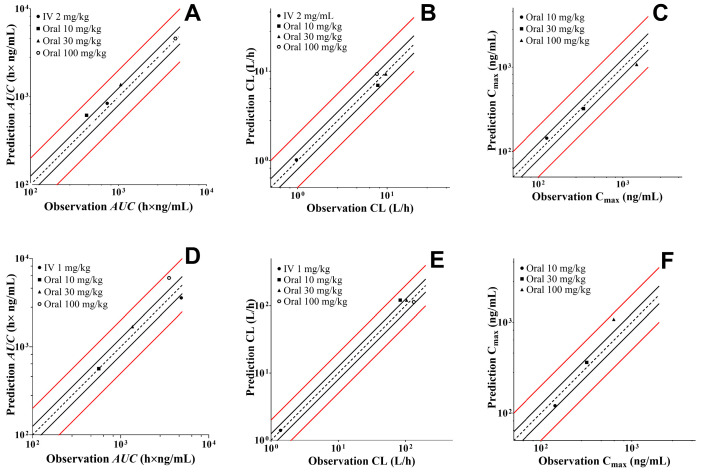
Predicted and validated pharmacokinetic results of SYHA1805 in SD rats and cynomolgus monkeys using the PBPK model. The black dashed lines represent the predicted values; the black solid lines indicate 80% and 125% of the predicted values; and the red solid lines denote 50% and 200% of the predicted values. (**A**–**C**) show the comparisons of AUC, CL, and Cmax in SD rats, respectively; (**D**–**F**) show the corresponding comparisons of AUC, CL, and C_max_ in cynomolgus monkeys.

**Figure 4:**
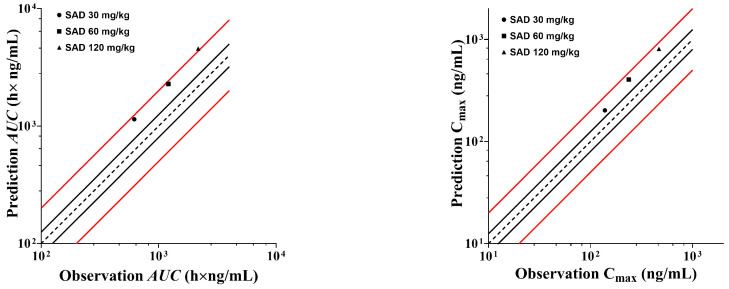
Predicted and validated pharmacokinetic results of SYHA1805 in human with PBPK model. The black dashed lines are the predicted values, the black solid lines are 80% and 125% of the predicted values, and the red solid lines are 50% and 200% of the predicted values.

The rat PBPK model was developed and validated using oral pharmacokinetic data at 10–100 mg/kg, with all predicted-to-observed pharmacokinetic ratios within 0.5–2.0-fold (Figure 3). Key parameters, including absorption rate constant (k_a_) and blood-to-plasma ratio (B/P), were subsequently translated to the monkey model, where species-specific clearance and distribution parameters were incorporated and successfully validated against both intravenous and oral data. Following cross-species validation, these parameters were extrapolated to humans, and the human PBPK model was constructed using allometrically scaled distribution and clearance parameters. Simulated human exposure profiles at clinically relevant doses are shown in Figure 4, and the PBPK model parameters and data sources are listed in Table 3.

**Figure 5:**
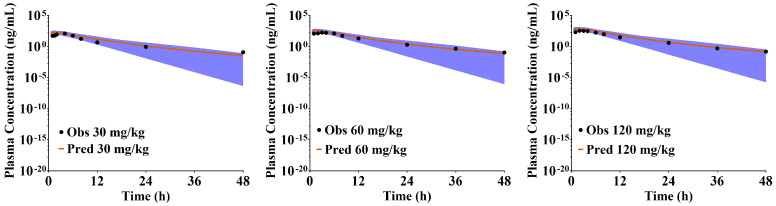
Predicted plasma concentration–time curve and 90% CI at different doses in human and the observed concentrations. The orange line is the predicted mean value at different times; the purple area is the 90% CI; the black dots are the observed mean concentrations at different times.

As described above, 30 mg was selected as the starting dose for the SAD phase of the FIH trial (CTR20202354). Blood samples were collected from all subjects at multiple time points from pre-dose to 72 h post-dose for drug concentration determination. Pharmacokinetic parameters were calculated and compared subsequently. As shown in Figure 5, predicted plasma concentration–time profiles were consistent with observed data, with most observations falling within the 90% confidence interval. Additionally, key pharmacokinetic parameters were within 0.5–2.0-fold of observed values, meeting predefined acceptance criteria and supporting the reliability of the translational PBPK framework.

## 4. Discussion

FXR agonists represent one of the most promising mechanistic classes for the treatment of MASH. However, clinical translation of FXR agonists has been limited by class-related adverse effects, including pruritus and gastrointestinal disturbances, which have constrained their therapeutic window. Accordingly, current efforts have increasingly focused on the development of next-generation FXR agonists with improved tissue or target selectivity to enhance safety while maintaining efficacy. Non-steroidal FXR agonists represent a particularly important direction in this regard, with candidates such as cilofexor, tropifexor, TERN-101, MET-409, vonafexor, and nidufexor currently comprising a major proportion of early-to-mid-stage clinical pipelines in this field [34]. In this context, SYHA1805 is also a potent non-steroidal FXR agonist with an EC_50_ of 13.85 nM and no detectable activity on TGR5 at concentrations up to 100 μM. Its potency is competitive with that of major non-steroidal FXR agonists currently in clinical development [34]. Moreover, broad off-target profiling in our preclinical study using the Safety47^TM^ platform (DiscoverX) demonstrated minimal activity across a wide range of GPCRs, nuclear receptors, transporters, ion channels, and kinases, supporting a highly selective pharmacological profile that may reduce the risk of off-target toxicity. This favorable selectivity profile strongly supports the further development of SYHA1805 as a promising therapeutic strategy for MASH.

Given the absence of approved FXR agonists for MASH and the known class-related safety liabilities, careful dose selection for FIH studies is critical. Inter-species extrapolation remains a cornerstone of early clinical dose prediction, and the integration of in vitro and in vivo ADME data can substantially improve the reliability of human pharmacokinetic prediction. In addition, PBPK modeling enables mechanistic integration of physiological and drug-specific parameters, providing a more robust framework for translational prediction than empirical scaling alone. Accordingly, we combined allometric scaling approaches with PBPK modeling to support FIH dose selection and to simulate human exposure profiles of SYHA1805.

SYHA1805 exhibited low oral bioavailability (~1% in cynomolgus monkeys), likely attributable to poor solubility, low intestinal permeability, and P-gp-mediated efflux. Although low bioavailability may necessitate higher oral doses to achieve therapeutic systemic exposure, this does not necessarily preclude clinical efficacy. In the SD rat tissue distribution study, SYHA1805 showed marked local enrichment in the gastrointestinal tract and liver following oral absorption, with predominant distribution to the liver post-absorption. The intestine and liver represent the primary sites of FXR expression [35]. Beyond the beneficial metabolic effects conferred by hepatic FXR activation, local intestinal FXR activation induces FGF15/19 secretion, which feeds back via the enterohepatic axis to suppress hepatic CYP7A1 expression and reduce bile acid synthesis [36]; this mechanism is analogous to FGF19-based therapeutic strategies for MASH [37]. Thus, despite limited systemic exposure, SYHA1805 may achieve sufficient local concentrations in key target organs to elicit clinical efficacy.

Systematic evaluation of metabolic stability and metabolite profiles across species indicated clear interspecies differences in biotransformation pathways. Among the evaluated species, cynomolgus monkeys demonstrated the closest resemblance to humans in terms of metabolic pathways and metabolite distribution, whereas beagle dogs showed similarity primarily at the level of metabolic stability in liver microsomes. Rodents, particularly rats, better reflected human-like metabolite patterns compared with mice. Collectively, these findings supported the selection of cynomolgus monkeys as the primary non-rodent species and rats as the representative rodent model for translational pharmacokinetic extrapolation.

Dose prediction is inherently sensitive to the selection of SF, particularly when human-relevant adverse effects cannot be fully captured in preclinical species. Although SYHA1805 did not induce mortality or irreversible toxicity in either SD rats or cynomolgus monkeys, dose-dependent alterations in liver-associated biochemical parameters (including ALP, cholesterol, HDL-C, and LDL-C) were observed. Importantly, class-associated adverse effects such as pruritus cannot be reliably assessed in animal models, introducing additional uncertainty in safety margin estimation. In addition, interspecies differences in pharmacokinetic behavior and low oral bioavailability further increased uncertainty in human exposure prediction. Considering these limitations collectively, a conservative safety factor of 100 was applied to ensure adequate safety margins for FIH dose selection.

Another challenge of this study lies in the selection of extrapolation parameters. In this study, we assumed that there were no species differences in the pharmacokinetics of SYHA1805 between rats and humans or between cynomolgus monkeys and humans, and that it exhibited linear pharmacokinetic characteristics within the studied dose range. However, significant differences were observed among species based on the PK results in rats and cynomolgus monkeys, especially with regards to gender differences in rats. To ensure the reliability of the extrapolation results, multiple allometric scaling models were used and CL_i.v,h_ was predicted for each species separately. The results showed significant differences among species, and even within rats, there were substantial differences obtained using different models. For the results, the lowest CL_i.v_ value was obtained using the *f_u_*-corrected intercept method, which may be attributed to the high plasma protein binding rate. The random errors introduced by sample preparation or instrument analysis will significantly affect the calculation results. On the other hand, when using the hepatic blood flow-corrected method, the predicted CL_i.v,h_ was extremely high. According to the equation, this difference may be due to the high systemic clearance rate in rats, which is approximately 10 times that of cynomolgus monkeys, and the value was not corrected by body weight using the above equation. To ensure the reliability of the results, we selected the extrapolated data from cynomolgus monkeys as the predicted CL_i.v,h_.

Additionally, there were also notable differences in predicted human V_ss_ using different species. From the in vitro incubation results, it was found that SYHA1805 exhibited poor stability in rat and mouse liver microsomes and hepatocytes, which may be the main reason for the significant differences in CL_i.v_ and V_ss_ in rats. Moreover, the stability results in rats differed greatly from those in humans, while the results in cynomolgus monkeys were more similar to human data. Furthermore, species-specific differences in plasma protein binding, tissue partitioning, and body composition may also contribute to this discrepancy [38]. Given the closer physiological and metabolic similarity between cynomolgus monkeys and humans, the monkey-derived V_ss_ of 15.1 L was selected as the central estimate for human PK projection, with the rat-derived value of 125 L retained as the upper bound to conservatively encompass cross-species variability.

Gender-dependent differences in both pharmacokinetics and toxicity were evident in rats, with female rats exhibiting higher systemic exposure and a greater incidence of pathological alterations than males following oral administration of SYHA1805. Such sex-based disparities in drug metabolism and transporters are well-documented [39,40,41] in rats and are often attributed to the sexually dimorphic secretion patterns of growth hormone, which regulate the expression of cytochrome P450 enzymes [42,43]. In contrast, no significant sex-related differences were observed in cynomolgus monkeys, suggesting that this phenomenon is species-specific. Despite the lack of sex differences in the non-rodent model, separate analyses for both sexes will be conducted in clinical trials to ensure comprehensive safety monitoring.

The PBPK modeling framework developed for SYHA1805 provided a translational bridge from preclinical species to humans, enabling prediction of systemic exposure and supporting FIH dose selection. A key uncertainty during model construction was the absence of experimentally determined B/P ratios across species, a parameter that can substantially influence predicted tissue distribution. In this context, the B/P ratio was indirectly inferred from intravenous PK data in rats and subsequently applied to cynomolgus monkeys and humans. While this approach enabled model completion in the absence of direct measurements, it necessarily assumes conserved blood partitioning across species. This simplification does not fully account for potential interspecies differences arising from plasma protein binding, erythrocyte partitioning, or hematocrit variability, and therefore represents a source of structural uncertainty in the model [44]. Similarly, oral absorption was constrained by limited biopharmaceutical data. The low oral bioavailability observed in both rats and monkeys suggests that SYHA1805 is characterized by poor intestinal absorption, likely driven by low permeability and/or solubility-limited uptake. To capture this behavior, a single first-order absorption rate constant was derived from rat oral pharmacokinetics and extrapolated to non-rodent species (cynomolgus monkeys and humans). Although this assumption was supported by comparable absorption trends between rats and monkeys, it may oversimplify species-specific and formulation-dependent absorption processes [45]. Despite these limitations, the PBPK model demonstrated acceptable predictive performance against observed preclinical and clinical PK data, supporting its utility for translational dose prediction. However, a systematic tendency to overpredict human exposure was observed when using a simplified first-order absorption framework. This discrepancy highlights the limitations of lumped absorption assumptions for compounds with low aqueous solubility and restricted permeability. Future model refinement should therefore incorporate mechanistic absorption components that explicitly describe dissolution, precipitation, and intestinal transit processes [33,45]. Such improvements are expected to reduce prediction bias and further enhance the reliability of PBPK-guided dose selection in early clinical development. Overall, a PBPK model for SYHA1805 was successfully established and validated using clinical data. This model represents a robust platform to inform the design and dose optimization of forthcoming clinical trials, including those evaluating multiple-dose regimens and drug–drug interaction potentials, ultimately accelerating the efficient clinical development of SYHA1805.

## 5. Conclusions

By combining in vitro and in vivo experiments, this study systematically determined the pharmacokinetic and toxicokinetic parameters of SYHA1805. These parameters were further employed to predict the FIH doses and pharmacokinetic behavior of SYHA1805 in a healthy Chinese population with the use of AS models and the PBPK model. The predicted doses were successfully applied in the following FIH clinical trials and the pharmacokinetic parameters were well simulated. The results of this study provide valuable support for the subsequent clinical development of this drug in a safer and more efficient manner.

## Figures and Tables

**Table 1:** Permeability test results of SYHA1805 in Caco-2 cells (n = 3).

Compound	+/− GF120918	Apparent Permeability Coefficient (A-B) (*P_app_*, ×10^−6^ cm/s)	Apparent Permeability Coefficient (B-A) (*P_app_*, ×10^−6^ cm/s)	Efflux Ratio (ER)
		*P_app_* (Mean ± SD)	*P_app_* (Mean ± SD)	
SYHA1805 (2 μM)	−	0.32 ± 0.04	8.15 ± 0.06	25.46
	+	1.32 ± 0.07	1.55 ± 0.04	1.17
SYHA1805 (10 μM)	−	0.33 ± 0.00	7.90 ± 0.12	23.66
	+	1.32 ± 0.06	2.46 ± 0.41	1.87
SYHA1805 (100 μM)	−	0.53 ± 0.05	10.31 ± 0.43	19.28
	+	1.17 ± 0.04	2.85 ± 0.25	2.43

**Table 2:** Average pharmacokinetic parameters in SD rats and cynomolgus monkeys after single administration of SYHA1805 (n = 6).

Species	Parameter	Group 1 I.V.	Group 2 PO (Low Dose)	Group 3 PO (Medium Dose)	Group 4 PO (High Dose)
SD rats	C_0_ or C_max_ (nM)	6420 ± 2610	126 ± 63.6	343 ± 139	1460 ± 256
	T_max_ (h)	-- ^2^	1.08 ± 0.736	0.958 ± 0.813	0.475 ± 0.137
	T_1/2_ (h)	1.11 ± 0.195	1.34 ± 0.250	2.27 ± 1.10	5.78 ± 2.68
	V_dss_ (L/kg)	1.78 ± 0.493	--	--	--
	CL (mL/min/kg)	80.9 ± 20.4	--	--	--
	AUC_0-last_ (nM·h)	744 ± 203	434 ± 232	1060 ± 371	4430 ± 1030
	AUC_0-inf_ (nM·h)	748 ± 205	448 ± 239	1090 ± 375	4450 ± 1020
	Bioavailability (%) ^1^	--	12.0	9.71	11.9
Cynomolgus monkeys	C_0_ or C_max_ (nM)	30,700 ± 8040	143 ± 73.4	319 ± 131	633 ± 346
	T_max_ (h)	--	2.08 ± 1.11	1.08 ± 0.492	1.50 ± 1.22
	T_1/2_ (h)	3.30 ± 1.60	3.16 ± 1.86	5.02 ± 1.21	4.27 ± 1.40
	V_dss_ (L/kg)	0.216 ± 0.109	--	--	--
	CL (mL/min/kg)	8.35 ± 2.20	--	--	--
	AUC_0-last_ (nM·h)	3580 ± 857	564 ± 351	1360 ± 709	3540 ± 942
	AUC_0-inf_ (nM·h)	3600 ± 853	669 ± 328	1440 ± 730	3640 ± 1000
	Bioavailability (%)	--	1.58	1.27	0.989

^1^ *AUC*_0-*last*_ and dose were used to calculate the bioavailability. ^2^ --: Not Applicable.

**Table 3:** PBPK model parameters and data sources of rat, monkey, and human.

Parameter	Route (Species)				
	I.V. (Rat)	Oral (Rat)	I.V. (Monkey)	Oral (Monkey)	Oral (Human)
log p	1.63	1.63	1.63	1.63	1.63
pk_a_	2.95	2.95	2.95	2.95	2.95
B/P	1.00	1.00	1.00	1.00	1.00
f_u_	0.011	0.011	0.002	0.002	0.003
Absorption Model	-- ^1^	First-order	--	First-order	First-order
F_a_	--	1.00	--	0.02	1.00
k_a_ (1/h)	--	0.30	--	0.30	0.30
F_u(Gut)_	--	1.00	--	1.00	1.00
Permeability Assay	--	Caco-2	--	Caco-2	Caco-2
P_Caco-2_ (10^−6^ cm/s)	--	0.39	--	0.39	0.39
Distribution Model	Full PBPK Model	Full PBPK Model	Full PBPK Model	Full PBPK Model	Full PBPK Model
V_ss_ input type	Predicted (1.85 L/kg)	Predicted (1.85 L/kg)	Predicted (0.23 L/kg)	Predicted (0.23 L/kg)	Predicted (0.21 L/kg)
Prediction Method	Method 2	Method 2	Method 2	Method 2	Method 2
log p_o:w_	1.63	1.63	1.63	1.63	1.63
K_p_ Scalar	19.00	19.00	3.00	3.00	3.00
CL_i.v_ (mL/min)	16.99	16.99	23.46	23.46	20.70

^1^ --: Not Applicable.

## Data Availability

The data presented in this study are available on request from the corresponding author.

## Supplementary Materials

The following supporting information can be downloaded at: https://www.mdpi.com/article/10.3390/pharmaceutics18070862/s1, Figure S1: Chemical structure of SYHA1805; Figure S2: Metabolic stability of SYHA1805 in liver microsomes from different species. The Y-axis represents the peak area ratio of SYHA1805 to the internal standard. n = 3 at each time point; Figure S3: Plasma concentration-time curves of SYHA1805 in SD rats and cynomolgus monkeys following intravenous and oral administration (n = 6 per dose group, with equal sex distribution); Figure S4: Tissue distribution profiles of SYHA1805 in male (upper panel) and female (lower panel) SD rats following a single oral dose of 30 mg/kg. (n = 6 per time point, with equal sex distribution); Table S1: Parameters used to calculate hepatic clearance; Table S2: Half-life, intrinsic clearance and hepatic clearance in different species liver microsomes (n = 3); Table S3: Metabolites identified in human, cynomolgus, beagle, rat and mouse liver microsome (n = 1); Table S4: Metabolites identified in human, cynomolgus, beagle, rat and mouse hepatocyte (n = 1); Table S5: Plasma protein binding of SYHA1805 in CD-1 mice, SD rats, beagle dogs, cynomolgus monkeys, and humans (n = 3); Table S6: Gender differences in systemic exposure after a single dose of SYHA1805 in SD rats (n = 6 per dose, equal sex distribution); Table S7: Predicted human CLi.v and Vss values from rat and cynomolgus data; Table S8: Predicted maximum recommended starting dose and maximum tolerated dose in first-in-human clinical trials.
