# Supplementary material for: First-in-Human Dose Selection, Pharmacokinetics Prediction, and Clinical Validation of SYHA1805, a Novel FXR Agonist, Using Allometric Scaling and PBPK Modeling
Source: Pharmaceutics. 2026 Jul 15;18(7):862. doi: 10.3390/pharmaceutics18070862 (PMC13414496; doi:10.3390/pharmaceutics18070862)
Supplement: Supplementary file 1 [file pharmaceutics-18-00862-s001.zip › pharmaceutics-4362851-supplementary.pdf]

1. Calculation of the percentage remaining, half-life ($T_{1/2}$), intrinsic clearance (CL_{int}), and hepatic clearance (CL_{hep}).

$$\%Remaining=\frac{Peak\ area\ ratio\ of\ reference\ to\ internal\ standard\ at\ any\ point}{Peak\ area\ ratio\ of\ reference\ to\ internal\ standard\ at\ 0\ min}\times 100 \quad \text{E.q.S1}$$

$$CL_{int(mic)}=\frac{0.693}{T_{1/2}\times Microsomal\ protein\ content} \quad \text{E.q.S2}$$

$$CL_{int(liver)} = CL_{int(mic)}\times Liver\ protein\ content(mg/g)\times Liver/Body\ weight$$

E.q.S3

$$CL_{hep}=\frac{CL_{int(liver)}\times Q_h}{CL_{int(liver)}+Q_h} \quad \text{E.q.S4}$$

Where $CL_{int(mic)}$ is the intrinsic clearance of liver microsomes, $CL_{int(liver)}$ is the intrinsic clearance of liver, CL_{hep} is the hepatic clearance, Q_h is the liver blood flow.

2. Calculation of the Papp and the efflux ratio in Caco-2 monolayer cell.

$$P_{app} = \frac{V_R}{Area\times Time}\times\frac{C_R}{C_0} \quad \text{E.q.S5}$$

$$ER = \frac{Papp\ (B-A)}{Papp\ (A-B)} \quad \text{E.q.S6}$$

Where V_R is the volume of the receiver solution (0.075 mL for side A and 0.25 mL for side B), *Area* is the relative surface area of the cell monolayer (0.0804 cm^2), *Time* is the incubation time (7200 s), C_0 is the initial concentration of the test compound in the donor compartment, and C_R is the final concentration in the receiver compartment.

3. Calculation of human intravenous clearance (CLi.v, h) and steady-state volume of distribution (Vss, h)

The calculation methods of $CL_{i.v}$ were as follows:

Single-species simple scaling method:

$$CL_{i.v,\ h}=a\times CL_{i.v,\ a}\times\frac{BW_h}{BW_a} \quad \text{E.q.S7}$$

Allometric scaling for a single species method:

$$CL_{i.v,\ h}=\frac{CL_{i.v,\ a}}{f_{u.\ a}}\times\frac{BW_h}{BW_a}^{0.75}\times f_{u,\ h} \quad \text{E.q.S8}$$

f_u corrected intercept method:

$$CL_{i.v,\ h}=33.35\times\frac{b}{Rf_{up}}^{0.77}\times mL/min \quad \text{E.q.S9}$$

Hepatic blood flow method:

$$CL_{i.v, h}=CL_{i.v, a}\times \frac{Q_h}{Q_a}$$ E.q.S10

Where $CL_{i.v, h}$ represents human intravenous clearance rate, $CL_{i.v, a}$ represents animal intravenous clearance rate, BW_h represents human body weight, BW_a represents animal body weight, and a represents the allometric scaling factor，for rats is 0.152, and for cynomolgus monkeys is 0.407. b represents the intercept, which is calculated based on the regression of $Ln(CL)$ and $Ln(WT)$ from different animal species experiments, in this study it was 20.77 as calculated. $f_{u, a}$ represents animal plasma free fraction, $f_{u, h}$ represents human plasma free fraction. Rf_{up} represents the ratio of animal plasma free fraction to human plasma free fraction. Q_h is the hepatic blood flow for humans, which is 1450 mL/min. Q_a is the hepatic blood flow for animals, which is 11.8 mL/min for rats and 122.5 mL/min for cynomolgus monkeys after correcting with actual body weight.

The calculation method of V_{ss} was as follows:

Allometric scaling method:

$$V_{ss, h}=a\times V_{ss, a}\times \frac{BW_h}{BW_a}$$ E.q.S11

Where $V_{ss, h}$ represents human steady-state apparent distribution volume, $V_{ss, a}$ represents animal steady-state apparent volume of distribution, BW_h represents human body weight, BW_a represents animal body weight, and a represents the allometric scaling factor. For rats is 0.75, and for cynomolgus monkeys is 0.86.

4. Calculation of Human Equivalent Dose (HED) for the clinical study of SYHA1805 in healthy individuals

Exposure-based method:

$$HED=AUC_{0\text{-}24h}\times \frac{f_{u,a}}{f_{u, h}}\times \frac{Cl_{i, v,h}}{F_a}$$ E.q.S12

$$MRSD=HED\times SF$$ E.q.S13

Where $AUC_{0\text{-}24h}$ represents animal steady-state AUC, $f_{u, a}$ represents animal plasma free fraction, $f_{u, h}$ represents human plasma free fraction, $CL_{i.v, h}$ represents human intravenous clearance rate, F_a represents animal bioavailability. SF is set as 100 in this study.

Body surface area-based method:

$$HED=NOAEL_a\times K\times BW_h$$ E.q.S14

Where $NOAEL_a$ represents animal No Observed Adverse Effect Level, K represents coefficient, for rat is 0.16 and for monkey is 0.32, BW_h represents human body weight.

Table S1. Parameters used to calculate hepatic clearance

Species	Liver weight/body weight (g/kg Body Weight)	Liver blood flow (Q_h) (mL/min/kg)	Microsome protein content (mg/g liver weight)

Mice	88	90.0	
Rat	40	55.2	
Beagle	32	30.9	45
Cynomolgus	30	43.6	
Human	20	20.7	

Table S2. Half-life, intrinsic clearance and hepatic clearance in different species liver microsomes (n=3)

Species	$T_{1/2}$ **(min)**	$CL_{int(mic)}$ **(μL/min/mg protein)**	$CL_{int(liver)}$ **(mL/min/kg)**
CD-1 mice	2.8	499.7	1978.9
SD rat	5.9	234.2	421.6
Beagle dog	65.0	21.3	30.7
Cynomolgus monkey	15.6	89.0	120.2
Human	50.4	27.5	24.8

Table S3. Metabolites identified in human, cynomolgus, beagle, rat and mouse liver microsome (n=1)

**Metabolite**	**Structural modification**	**Metabolic pathway**	**Relative abundance（UV %）**				
			human	cynomolgus	beagle	rat	mice
M3	P + O – Cl + $C_{10}H_{15}N_3O_6S$	Mono-oxidation, dechlorination, and glutathione conjugation	ND	ND	ND	ND	2.55
M4	P + O – Cl + $C_{10}H_{15}N_3O_6S$	Mono-oxidation, dechlorination, and glutathione conjugation	ND	ND	ND	ND	2.95
M7	P + O – Cl + $C_{10}H_{15}N_3O_6S$	Mono-oxidation, dechlorination, and glutathione conjugation	ND	ND	ND	ND	2.33
M10	P + $C_{10}H_{15}N_3O_6S$	Glutathione conjugation	ND	ND	ND	3.01	6.55
M11	P + 2O – Cl	Di-oxidation and dechlorination	ND	ND	ND	2.89	ND
M13	P + 2O + 2H	Di-oxidation and hydrogenation	44.29	40.01	19.17	16.02	7.05
M14	P + O - 2H	Mono-oxidation and dehydrogenation	ND	ND	ND	+	5.55
M15	P + 2O - Cl	Di-oxidation and dechlorination	+	1.37	ND	+	ND
M16	P + 2O	Di-oxidation	2.39	5.94	0.7	15.83	7.43
M18	P + O	Mono-oxidation	7.13	5.83	2.72	35.8	28.04
M19	P + O	Mono-oxidation	+	+	ND	2.19	1.01
M20	P- 2H	Dehydrogenation	+	+	+	ND	8.74
SYHA1805	NA	-	46.19	46.86	77.41	24.27	27.8
Total	NA	-	100	100	100	100	100

[1] The table presents semi-quantitative data obtained from the peak area of UV (254-340 nm) in the chromatogram. ND: Not detected; NA: Not applicable; P: Parent drug; +: Detected in mass spectrometry, but the UV peak at 254-340 nm cannot be integrated.

Table S4. Metabolites identified in human, cynomolgus, beagle, rat and mouse hepatocyte (n=1)

Metabolite	Structural modification	Metabolic pathway	Relative abundance（UV %）				
			human	cynomolgus	beagle	rat	mice
M1	P + 2O – Cl + $C_{10}H_{15}N_3O_6S$	Di-oxidation, dechlorination, and glutathione conjugation	ND	ND	ND	ND	2.75
M2	P + 2O – Cl + $C_{10}H_{15}N_3O_6S$	Di-oxidation, dechlorination, and glutathione conjugation	ND	ND	ND	ND	2.1
M3	P + O – Cl + $C_{10}H_{15}N_3O_6S$	Mono-oxidation, dechlorination, and glutathione conjugation	ND	ND	ND	12.28	11.04
M4	P + O – Cl + $C_{10}H_{15}N_3O_6S$	Mono-oxidation, dechlorination, and glutathione conjugation	0.69	0.47	+	11.11	14.59*
M5	P + O + $C_{10}H_{15}N_3O_6S$	Mono-oxidation and glutathione conjugation	ND	ND	ND	+	12.38*
M6	P + O + 2H + $C_{10}H_{15}N_3O_6S$	Mono-oxidation, hydrogenation, and glutathione conjugation	ND	ND	2.06	23.28	ND
M7	P + O – Cl + $C_{10}H_{15}N_3O_6S$	Mono-oxidation, dechlorination, and glutathione conjugation	3.28	5.46	+	+	2.48
M8	P + $C_{10}H_{15}N_3O_6S$	Glutathione conjugation	ND	ND	+	3.98	3.75
M9	P + $C_{10}H_{15}N_3O_6S$	Glutathione conjugation	ND	ND	+	6	5.14
M10	P + $C_{10}H_{15}N_3O_6S$	Glutathione conjugation	ND	ND	4.03	37.06	41.8
M12	P + O	Mono-oxidation	ND	+	+	5.33	+
M13	P + 2O + 2H	Di-oxidation and hydrogenation	42.85	44.05	8.36	ND	ND
M17	P + O	Mono-oxidation	ND	ND	8.86	ND	ND
M18	P + O	Mono-oxidation	2.64	+	+	ND	ND
M20	P- 2H	Dehydrogenation	0.38	+	+	ND	1.45
SYHA1805	NA	-	50.17	50.01	76.68	0.96	2.53
Total	NA	-	100	100	100	100	100

[1] The table presents semi-quantitative data obtained from the peak area of UV (254-340 nm) in the chromatogram. ND: Not detected; NA: Not applicable; +: Detected in LC-MS, but the UV peak signal at 254-340 nm is too weak to integrate the peak area; *: UV peak area cannot be separately integrated, data given based on the ratio of mass spectrometry peak area.

Table S5. Plasma protein binding of SYHA1805 in CD-1 mice, SD rats, beagle dogs, cynomolgus monkeys, and humans (n=3)

Species	Concentration (μM)	Unbound (%) (n=3)	Bound (%) (n=3)
CD-1 Mouse	0.2	<1	>99.0
	2	0.4	99.6
	10	0.9	99.1
Sprague-Dawley Rat	0.2	<1	>99.0
	2	0.5 ± 0.1	99.5
	10	1.6	98.4
Beagle dog	0.2	<1	>99.0
	2	0.5	99.5
	10	0.5	99.5
Cynomolgus monkey	0.2	<1.1	>98.9
	2	<0.1	>99.9
	10	0.2	99.8
Human	0.2	<1	>99.0
	2	0.2 ± 0.2	99.8
	10	0.4	99.6

Table S6. Gender differences in systemic exposure after a single dose of SYHA1805 in SD rats (n=6 per dose, equal sex distribution)

Route of medication	*Dose* (mg/kg)	C_0 or C_{max} (female/male)	Ratio of C_0 or C_{max} (female/male)	$AUC_{0\text{-}last}$ (female/male)	Ratio of $AUC_{0\text{-}last}$ (female/male)
**Intravenous injection**	2.00	4270/8570	0.498	596/891	0.669
**Oral administration**	10.0	167/85.7	1.95	599/269	2.23
**Oral administration**	30.0	418/268	1.56	1310/804	1.63
**Oral administration**	100	1490/1420	1.05	5120/3730	1.37

Table S7. Predicted human $CL_{i.v}$ and V_{ss} values from rat and cynomolgus data

Item	Prediction method	Result
$CL_{i.v}$ (L/h)	Single-species scaling approaches (rat)	51.6
	Allometric scaling (rat)	22.8
	fu Corrected Intercept Method (rat)	7.9
	Hepatic Blood Flow Corrected Method (rat)	127.5
	**Mean (rat)**	**52.5**
	Single-species scaling approaches (cynomolgus)	14.3
	Allometric scaling (cynomolgus)	23.5
	fu Corrected Intercept Method (cynomolgus)	28.3
	Hepatic Blood Flow Corrected Method (cynomolgus)	16.7
	**Mean (cynomolgus)**	**20.7**
V_{ss} (L)	Allometric scaling (rat)	125
	Allometric scaling (cynomolgus)	15.1

Table S8. Predicted maximum recommended starting dose and maximum tolerated dose in first in human clinical trials

Species	Method	NOAEL (mg/kg/day)	Steady AUC_{0-24} (h×nM)	Human equivalent AUC_{0-24} (ng×h/mL)	HED (mg)	SF	MRSD (mg)
Rat	Exposure-based method	1000	19687	40297	17630	100	176
	Body surface area-based method				11200	100	112
Monkey	Exposure-based method	150	23725	9250	12118	100	121
	Body surface area-based method				3360	100	33

[1] NOALE: No Observed Adverse Effect Level; HED: Human Equivalent Dose; SF: Safety factor; MRSD: maximum recommended starting dose

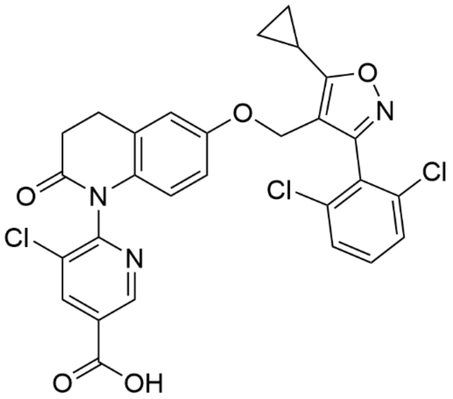

Figure S1. Chemical structure of SYHA1805.

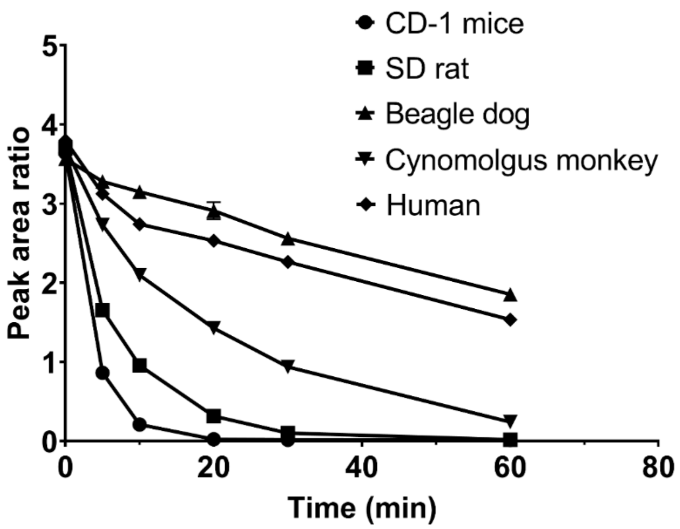

Figure S2. Metabolic stability of SYHA1805 in liver microsomes from different species. The Y-axis represents the peak area ratio of SYHA1805 to the internal standard. n = 3 at each time point.

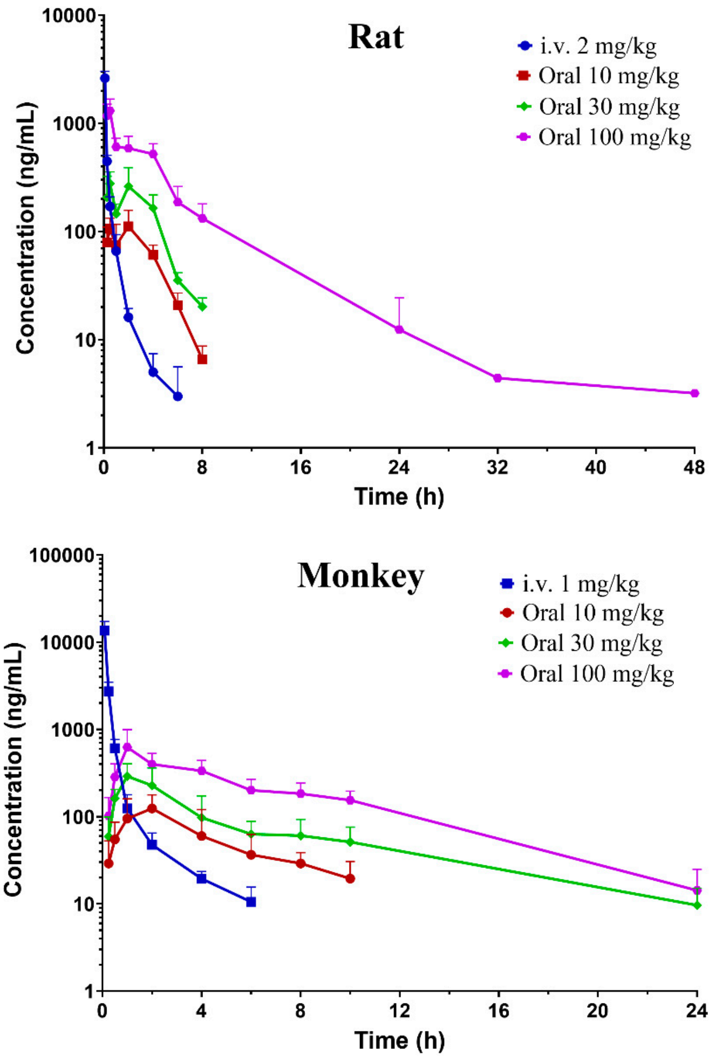

Figure S3. Plasma concentration-time curves of SYHA1805 in SD rats and cynomolgus monkeys following intravenous and oral administration (n = 6 per dose group, with equal sex distribution).

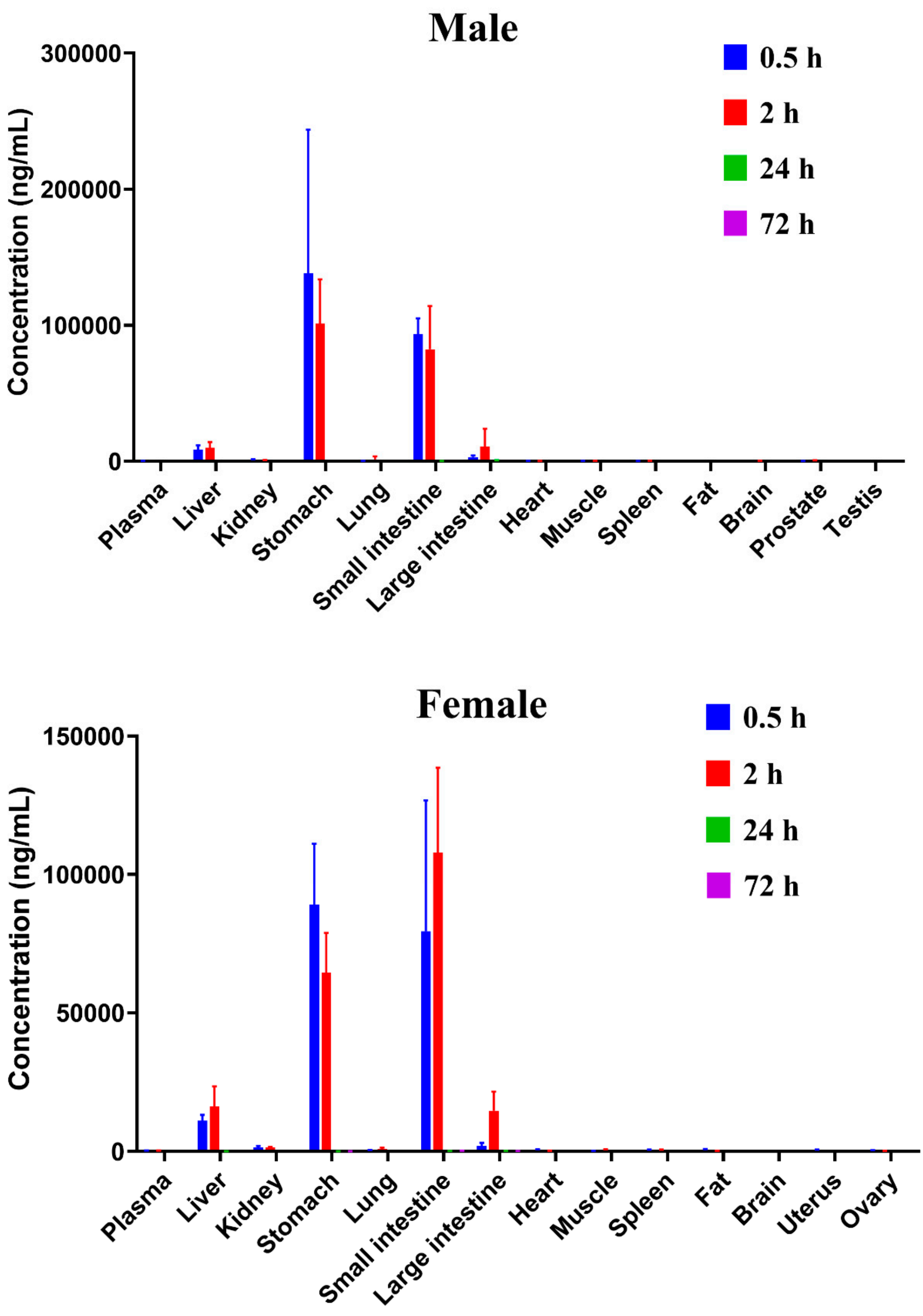

Figure S4. Tissue distribution profiles of SYHA1805 in male (upper panel) and female (lower panel) SD rats following a single oral dose of 30 mg/kg. (n = 6 per time point, with equal sex distribution)
